# The crosstalk between lncRNAs and the Hippo signalling pathway in cancer progression

**DOI:** 10.1111/cpr.12887

**Published:** 2020-08-10

**Authors:** Chao Tu, Kexin Yang, Lu Wan, Jieyu He, Lin Qi, Wanchun Wang, Qiong Lu, Zhihong Li

**Affiliations:** ^1^ Department of Orthopedics The Second Xiangya Hospital Central South University Changsha China; ^2^ Hunan Key Laboratory of Tumor Models and Individualized Medicine The Second Xiangya Hospital Central South University Changsha China; ^3^ Xiangya School of Medicine Central South University Changsha China; ^4^ Department of Geriatrics The Second Xiangya Hospital Central South University Changsha China; ^5^ Department of Pharmacy The Second Xiangya Hospital Central South University Changsha China

**Keywords:** cancer, crosstalk, Hippo, LncRNA, sarcoma, YAP

## Abstract

LncRNAs play a pivotal role in the regulation of epigenetic modification, cell cycle, differentiation, proliferation, migration and other physiological activities. In particular, considerable studies have shown that the aberrant expression and dysregulation of lncRNAs are widely implicated in cancer initiation and progression by acting as tumour promoters or suppressors. Hippo signalling pathway has attracted researchers’ attention as one of the critical cancer‐related pathways in recent years. Increasing evidences have demonstrated that lncRNAs could interact with Hippo cascade and thereby contribute to acquisition of multiple malignant hallmarks, including proliferation, metastasis, relapse and resistance to anti‐cancer treatment. Specifically, Hippo signalling pathway is reported to modulate or be regulated by widespread lncRNAs. Intriguingly, certain lncRNAs could form a reciprocal feedback loop with Hippo signalling. More speculatively, lncRNAs related to Hippo pathway have been poised to become important putative biomarkers and therapeutic targets in human cancers. Herein, this review focuses on the crosstalk between lncRNAs and Hippo pathway in carcinogenesis, summarizes the comprehensive role of Hippo‐related lncRNAs in tumour progression and depicts their clinical diagnostic, prognostic or therapeutic potentials in tumours.

## Graphical abstract

Increasing evidences have demonstrated that lncRNAs could interact with Hippo signalling cascade and thereby contribute to acquisition of multiple malignant hallmarks, including proliferation, metastasis, relapse and resistance to anti‐cancer treatment. Specifically, Hippo signalling pathway is reported to modulate or be regulated by widespread lncRNAs. Intriguingly, certain lncRNAs could form a reciprocal feedback loop with Hippo signalling. More speculatively, lncRNAs related to Hippo pathway have been poised to become important putative biomarkers and therapeutic targets in human cancers.

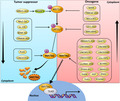

## 1 INTRODUCTION

Cancer is one of the life‐threatening diseases and remains a critical public health issue worldwide.^1^, ^2^, ^3^ Despite the tremendous improvements in cancer therapy in recent decades, there are still many patients who suffer from unsatisfactory outcomes.^4^ Currently, the underlying molecular mechanisms in tumour occurrence and progression have not yet been fully elucidated.^5^, ^6^ Meanwhile, efficient biomarkers for early diagnosis, prognosis prediction and therapeutic targets are still lacking, which may hinder the effective monitoring as well as treatment of cancer.^5^, ^7^, ^8^

Long non‐coding RNAs (lncRNAs) are a large and heterogeneous class of endogenous lncRNA family that are generally greater than 200 nucleotides (nts) in length.^9^ Previously, lncRNAs were characterized as transcriptional noise since they exhibit no or limited protein‐coding capacity.^10^, ^11^ Recently, owing to the advancement of next‐generation sequencing‐based transcriptome profiling, tremendous lncRNAs were identified and functionally annotated.^7^, ^12^, ^13^ LncRNAs are found to execute a wide spectrum of biological processes,^14^ such as alternative splicing, chromatin modification, sponging microRNAs (miRNAs) as competing endogenous RNA (ceRNAs), nuclear‐cytoplasmic trafficking or interaction with genes, and thereby involve in crucial regulation of various human diseases including cancer.^15^ Compelling experimental evidences indicate an engagement of lncRNAs in pleiotropic pathophysiological functions related to tumorigenesis, like the cell growth, invasion, metastasis, apoptosis and chemo‐resistance,^16^ by interaction with other macromolecules.^17^ Accumulating studies have shown that lncRNAs could be considered as a promising candidate in cancer prognosis and diagnosis.^1^, ^4^, ^7^, ^18^ Accordingly, lncRNAs have attracted great attention due to their multifaceted modulatory functions and the capacity as predictive biomarkers in cancers.^1^, ^4^, ^7^

Hippo signalling pathway consists of a broad range of proteins and controls lots of molecular and cellular processes.^12^ It is reported that Hippo pathway could be activated or suppressed by genetic or epigenetic regulation, leading to a plethora of pathological disorders including cancers.^11^ Notably, advanced studies have demonstrated that the crosstalk between lncRNAs and Hippo pathway may contribute to cancer occurrence and progression in recent years. For instance, YAP (or YAP1), a major transducer in downstream of Hippo pathway, is amplified and nuclear accumulated in a variety of cancers.^19^ LncRNA TNRC6C antisense RNA 1 (TNRC6C‐AS1) was reported to be abundantly expressed in thyroid carcinoma and could regulate the subcellular localization and activation of YAP, leading to the promotion of cell proliferation and tumorigenicity.^20^

In this review, we systematically summarize the up‐to‐date insights provided by studies regarding the crosstalk between lncRNAs and Hippo signalling pathways in cancers. In addition, we provide a brief overview of the Hippo‐related lncRNAs as clinicopathological biomarkers and highlight its potential role as therapeutic targets in cancers. The interplay between Hippo and lncRNA may shed light on the role of underlying mechanisms in carcinogenesis.

## 2 CANONICAL HIPPO SIGNALLING PATHWAY IN TUMORIGENESIS

The Hippo signalling pathway is initially characterized as a critical signalling cascade that regulates organ size in fruit fly (*drosophila melanogaster*) in 1995.^21^ It is an evolutionarily ancient and conserved network among different species,^22^ and its homology molecules in mammals have been subsequently identified. A growing number of studies have highlighted a critical role of Hippo pathway in the regulation of organ size, tissue homeostasis, cell proliferation, apoptosis, metastasis, autophagy, angiogenesis and stem cell self‐renewal.^23^, ^24^ The misregulation of Hippo signalling pathway can cause many disease conditions.^25^ In tumorigenesis, Hippo pathway is well‐established as a tumour‐suppressive cascade due to its proliferation restriction and apoptosis induction.^26^, ^27^

In mammals, the central axis of the Hippo signalling pathway comprises two serine/threonine kinases: mammalian sterile 20‐like kinase 1/2 (MST1/2) and its homolog large tumour suppressor 1 and 2 (LAST1/2); two adaptor/scaffold protein: WW45 for MST1/2 and Mps one binder kinase activator‐like 1 (MOB1) for LAST1/2; downstream transcriptional co‐regulators: yes‐associated protein (YAP) and its paralog transcriptional co‐activator with PDZ‐binding motif (TAZ, also known as WWTR1); and various nuclear transcriptional factors: transcriptional enhancer‐associated domain (TEAD1/2/3/4).^26^, ^28^ Of them, YAP and TAZ are key intracellular messengers, whose localizations are critical in Hippo pathway.^11^ YAP/TAZ could be positively or negatively modulated by phosphorylation at different sites by upstream kinases, elicit target gene expression signature through forming complexes with TEAD family, the major nuclear partner, and thereby play a prominent role in cellular plasticity, lineage differentiation during development, tumour initiation, progression and metastasis.^29^, ^30^

**Figure 1:**
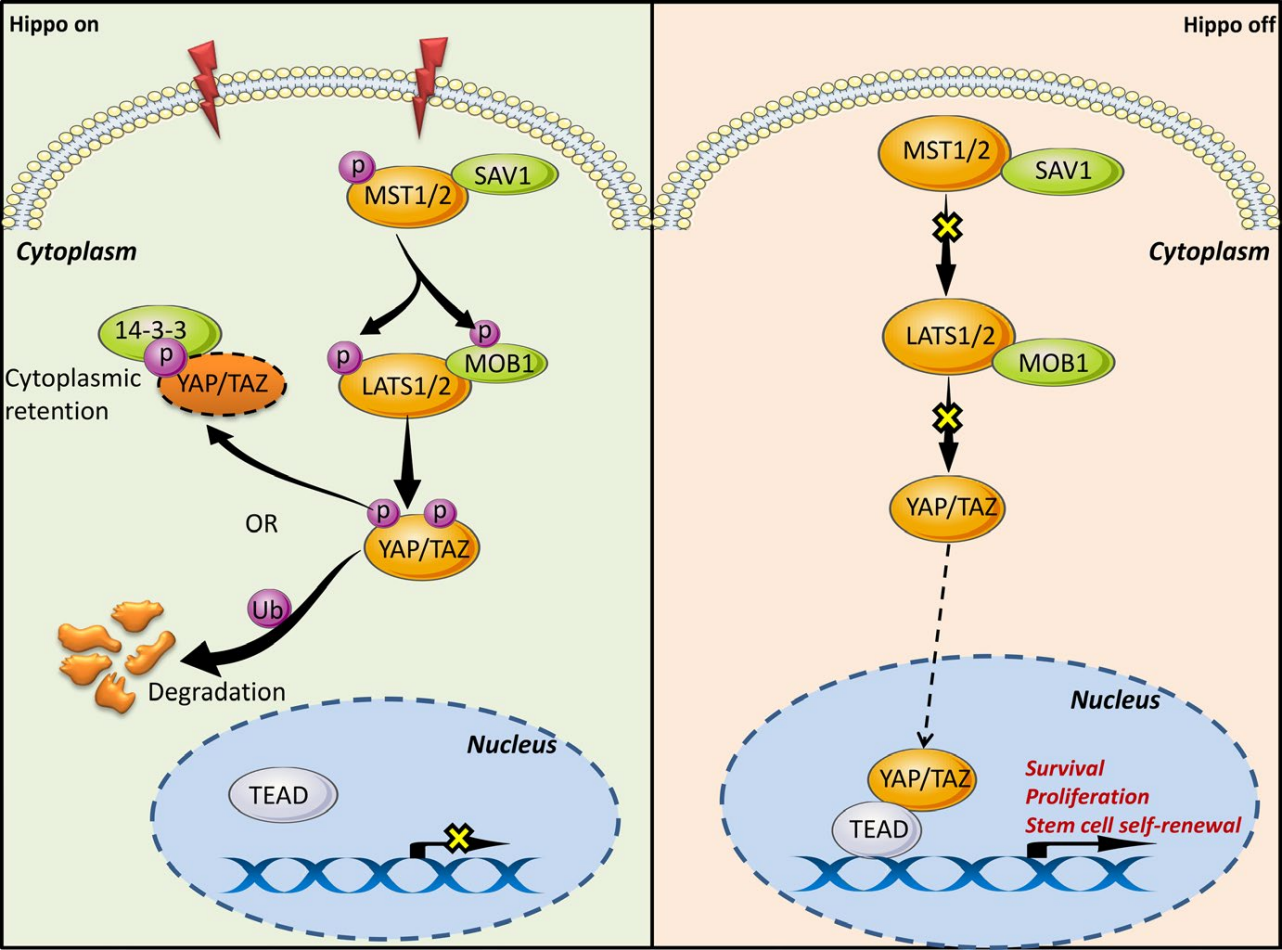
Molecular schematic of canonical Hippo signalling cascade in cancers

In canonical Hippo signalling, the cascade is on (‘Hippo On’) when the upstream Hippo pathway is activated by stimuli or regulators, such as mechanical stress, cell polarity determinants and increased cell‐cell contact.^28^, ^31^ Then, MST1/2 kinase is phosphorylated and subsequently phosphorylates salvador homolog 1 (SAV1) to form a heterotetramer to further promote the LATS1/2 phosphorylation. Activated LATS1/2 could result in inactivation of YAP/TAZ through sequestering its cytoplasmic localization by binding to 14‐3‐3 protein or degradation via ubiquitination, and thereby dampen the transcription of downstream genes.^11^, ^31^ Conversely, when the Hippo pathway is inactivated (‘Hippo off’), YAP/TAZ translocates to the nucleus and binds primarily to enhancer elements by using TEAD as DNA‐binding sites,^11^, ^32^ thereby driving target gene (*AREG*, *CTGF*, *Cyr61*, *ANKRD1*, *AXL*, etc) transcription and promoting cell tissue growth, survival, proliferation and self‐renewal,^28^, ^33^, ^34^ as presented in Figure 1.

## 3 REGULATORY NETWORK OF LNCRNAS AND HIPPO SIGNALLING PATHWAY IN CANCER

Overall, considerable crosstalk between lncRNAs and Hippo signalling pathway has been revealed in several tumours as demonstrated in Tables 1, 2, 3. A vast majority of lncRNAs were discovered in the regulation of Hippo signalling pathways. Conversely, Hippo pathways were also reported to modulate expression of lncRNAs.^31^ These bilateral regulations ultimately impact target gene expressions in cancer progression, indicating a close relationship and complexity between lncRNAs and Hippo signalling cascades.

**Table 1:** Overview of lncRNAs that regulate Hippo signalling pathway in cancer development

LncRNA	Tumour type	Expression	Interaction with Hippo cascade	Biological function in cancers	Ref.
B4GALT1‐AS1	Osteosarcoma, colon cancer	↑	B4GALT1‐AS1 directly or indirectly binds to YAP to promote its transcription	Proliferation, migration, spheroid formation, stemness, chemo‐resistance	35, 38
BCYRN1	Glioma	↑	BCYRN1 increases TAZ expression	Proliferation, invasion, migration	^40^
BDNF‐AS	Glioblastoma	↓	BDNF‐AS increases LATS1 and YAP phosphorylation mediated by RAX2/ DLG5	Proliferation, apoptosis, migration, invasion	^126^
FRMD6‐AS2	Endometrial cancer	↓	FRMD6‐AS2 increases phosphorylation of LATS1 and YAP	Tumour growth, migration and invasion	^127^
GHET1	NSCLC	↑	GHET1 enhances YAP expression	Proliferation, invasion, EMT	^36^
MAYA	Breast cancer bone metastasis	↑	MAYA induces inhibitory methylation of MST1	Bone metastasis of cancer cells	^128^
LEF1‐AS1	OSCC	↑	LEF1‐AS1 inhibits the binding of LATS1 to MOB, and thus suppresses Hippo pathway	Cell survival, proliferation, migration, apoptosis, cell cycle	^129^
LINC00174	CRC	↑	LINC00174 sponges to miR‐1910‐3p to activate TAZ	Cell growth	^32^
LINC00662	GC	↑	LINC00662 sponges to miR‐497‐5p to promote YAP expression	Proliferation, chemo‐sensitivity	^130^
LINC00673	BC	↑	LINC00673 increases MAPK4 and YAP/TAZ expression and reduces YAP phosphorylation	Proliferation, apoptosis, cell cycle	^131^
LINC01048	CSCC	↑	LINC01048 interacts with TAF15 to upregulate YAP	Proliferation, apoptosis	^132^
LINC01314	HB	↓	LINC01314 inhibits nuclear translocation of YAP	Proliferation, migration, cell cycle	^133^
LINC01559	Pancreatic cancer	↑	LINC01559 hinders YAP phosphorylation and enhances its transcription	Proliferation, migration, cell growth	^134^
Linc‐OIP5	BC, glioma	↑	Linc‐OIP5 increases YAP expression	Proliferation, migration, invasion, apoptosis, tube formation capacity	135, 136, 137
LncRNA‐ATB	HCC	↑	LncRNA‐ATB activates YAP expression	Cell proliferation, clonogenicity, autophagy	^95^
MIR100HG	Osteosarcoma	↑	MIR100HG silences LATS1/2 and inactivates Hippo	Proliferation, apoptosis, cell cycle	^42^
MRVI1‐AS1	NPC	↓	MRVI1‐AS1 promotes RASSF1 expression to suppress TAZ expression	Paclitaxel‐resistant	^46^
Nkx2‐2as	MB	↓	Nkx2‐2as upregulates LATS1/2	Cell division, migration	^138^
NSCLCAT1	NSCLC	↑	NSCLCAT1 represses MST1 and LATS1 and increases YAP/TAZ expression	Cell viability, migration, apoptosis, invasion	^139^
PCGEM1	Ovarian carcinoma	↑	PCGEM1 upregulates YAP expression	Proliferation, apoptosis, migration, invasion	^122^
PLK4	HCC	↓	PLK4 inactivates YAP and induces cell senescence	Cell viability, growth, cellular senescence	^118^
SNHG15	PTC	↑	SNHG15 upregulates YAP expression	Proliferation, apoptosis, migration, EMT	^103^
THOR	NPC	↑	THOR enhances YAP transcriptional activity	Proliferation, migration, invasion, spheres formation, stemness, cisplatin sensitivity	^140^
TNRC6C‐AS1	Thyroid carcinoma	↑	TNRC6C‐AS1 regulates MST1 and LATS1/2, and phosphorylation of YAP	Proliferation, apoptosis, autophagy	^20^
TUG1	RCC	↑	TUG1 enhances YAP expression	Proliferation, migration	^141^
uc.134	HCC	↓	uc.134 inhibits CUL4A‐mediated ubiquitination of LATS1 and increases YAP phosphorylation	Proliferation, invasion, metastasis	^96^
XIST	Osteosarcoma	↑	XIST increases YAP expression	Proliferation, invasion	^37^
ZFAS1	Prostate cancer	↑	ZFAS1 upregulates YAP and TEAD1 expression	Proliferation, invasion, EMT	^91^
ZFHX4‐AS1	BC	↑	ZFHX4‐AS1 increases YAP/TAZ expression	Proliferation, migration, apoptosis, invasion, cell cycle	^142^

Abbreviations: ↑ upregulated; ↓ downregulated; ATF3, activating transcription factor 3; B4GALT1‐AS1, B4GALT1 antisense RNA 1; BC, breast cancer; BCYRN1, brain cytoplasmic RNA 1; BDNF‐AS, BDNF antisense RNA; CRC, colorectal cancer; CSCC, cutaneous squamous cell carcinoma; DLG5, discs large homolog 5; EMT, epithelial‐to‐mesenchymal transition; FRMD6‐AS2, FRMD6 antisense RNA 2; GC, gastric cancer; GHET1, gastric cancer high expressed transcript 1; HB, hepatoblastoma; HCC, hepatocellular carcinoma; LATS1/2, large tumour suppressor homolog 1/2; LEF1‐AS1, LEF1 antisense RNA 1; Linc‐OIP5, linc‐Opa interacting protein 5; LncRNA‐ATB, lncRNA activated by transforming growth factor‐β; LSCC, laryngeal squamous cell carcinoma; MB, medulloblastoma; MIR100HG, mir‐100‐let‐7a‐2‐mir‐125b‐1 cluster host gene; MOB1, Mps one binder kinase activator‐like 1; MRVI1‐AS1, murine retrovirus integration site 1 homolog antisense RNA 1; MST1/2, mammalian sterile twenty‐like 1/2; NPC, nasopharyngeal carcinoma; NSCLC, non‐small‐cell lung cancer; NSCLCAT1, non‐small‐cell lung cancer‐associated transcript‐1; OSCC, oral squamous cell carcinoma; PCGEM1, prostate cancer gene expression marker 1; PDAC, pancreatic ductal adenocarcinoma; PLK4, polo‐like kinase 4; PTC, papillary thyroid carcinoma; RASSF1, ras‐associated domain family member 1; RCC, renal cell carcinoma; SNHG 15, small nucleolar RNA host gene 15; TAZ, transcriptional co‐activator with PDZ‐binding motif; TEAD, transcriptional enhancer‐associated domain; THOR, testis‐associated highly conserved oncogenic long non‐coding RNA; TNRC6C‐AS1, TNRC6C antisense RNA 1; TUG1, taurine upregulated gene 1; XIST, X‐inactive specific transcript; YAP, yes‐associated protein.

**Table 2:** Overview of Hippo signalling pathway induced lncRNAs in cancer development

LncRNA	Tumour type	Expression	Interaction with Hippo cascade	Biological function in cancers	Ref.
BCAR4	BC	↑	YAP promotes BCAR4 expression	Glycolysis	^26^
CYTOR (LINC00152)	CRC	↑	YAP increases CYTOR expression, which in turn sponges to miR‐632 and miR‐185‐3p to target FSCN1	Proliferation, invasion, metastasis	^51^
H19	Osteosarcoma, bladder cancer	↑	YAP increases H19 expression	Proliferation, migration	53, 90
MT1DP	Liver cancer	↓	YAP and Runx2 inhibit MT1DP expression dependent on FoxA1	Proliferation, apoptosis, colony formation	^47^
NORAD	Lung and breast cancer metastasis	↓	YAP/TAZ‐TEAD and NuRD complex repress NORAD expression	Migration and invasion	^52^

Abbreviations: ↑ upregulated; ↓ downregulated; BC, breast cancer; BCAR4, breast cancer antiestrogen resistance 4; CRC, colorectal cancer; CYTOR, cytoskeleton regulator RNA; FSCN1, fascin actin‐binding protein 1; MT1DP, metallothionein 1D, pseudogene; NORAD, non‐coding RNA activated by DNA damage; TAZ, transcriptional co‐activator with PDZ‐binding motif; TEAD, transcriptional enhancer‐associated domain; YAP, yes‐associated protein.

**Table 3:** Overview of lncRNAs that form reciprocal interactions with Hippo pathway in cancer development

LncRNA	Tumour type	Expression	Interaction with Hippo cascade	Biological function in cancers	Ref.
GAS5	Pancreatic cancer, CRC	↓	GAS5 enhances cytoplasm translocation of YAP and promotes phosphorylation and ubiquitin‐mediated YAP degradation. YAP could target YTHDF3, which reversibly bound m^6^A‐methylated GAS5 to facilitate its decay.	Cell viability, chemo‐resistance	11, 65
LINC01433	GC	↑	LINC01433 decreases YAP phosphorylation, and YAP activates LINC01433 transcription	Proliferation, migration, invasion, chemo‐resistance	^12^
LncARSR	RCC	↑	LncARSR inhibits LATS/YAP interaction to facilitate YAP nuclear translocation, which in turn transactivates lnARSR expression	Renal tumour‐initiating cell self‐renewal, tumorigenicity and metastasis	^143^
MALAT1	MM, CRC, pancreatic cancer, liver cancer, Breast cancer,	↑ ↓	MALAT1 directly decreases LATS to increase YAP activity or sponges to miR‐181a‐5p to target YAP. YAP attenuates the nuclear retention of SRSF1 and abrogates its inhibitory effect on MALAT1. Besides, MALAT1 sequesters TEAD and blocks YAP‐TEAD binding	Proliferation, apoptosis, cell adhesion, angiogenesis, migration, invasion, cancer metastasis, EMT	66, 67, 68, 81, 92
SNHG1	LSCC	↑	SNHG1 sponges to miR‐375 to increase YAP expression, and YAP activates SNHG1 transcription	Proliferation, migration, invasion, apoptosis	^84^
THAP9‐AS1	PDAC	↑	THAP9‐AS1 sponges to miR‐484 to indirectly enhance YAP activity, or directly bind to YAP, and in turn inhibit the dephosphorylation of YAP. Besides, YAP/TEAD1 promotes THAP9‐AS1 transcription	Cell growth	^85^
UCA1	Pancreatic cancer, thyroid cancer, ovarian cancer	↑	UCA1 enhances AMOT‐YAP interaction to promote YAP nuclear translocation. Increased YAP promotes UCA1 transcription	Proliferation, apoptosis, migration, invasion, EMT	61, 62, 63, 64, 144

Abbreviations: ↑ upregulated; ↓ downregulated; AMOT, angiomotin; CRC, colorectal cancer; GAS5, growth arrest‐specific 5; GC, gastric cancer; LSCC, laryngeal squamous cell carcinoma; MALAT1, metastasis‐associated lung adenocarcinoma transcript 1; MM, multiple myeloma; MOB1, Mps one binder kinase activator‐like 1; PDAC, pancreatic ductal adenocarcinoma; RCC, renal cell carcinoma; SNHG1, small nucleolar RNA host gene 1; SRSF1, serine‐/arginine‐rich splicing factor 1; TAZ, transcriptional co‐activator with PDZ‐binding motif; TEAD, transcriptional enhancer‐associated domain; THAP9‐AS1, THAP9 antisense RNA 1; UCA1, urothelial cancer‐associated 1; YAP, yes‐associated protein.

### 3.1 LncRNAs regulate members of Hippo pathway

**Figure 2:**
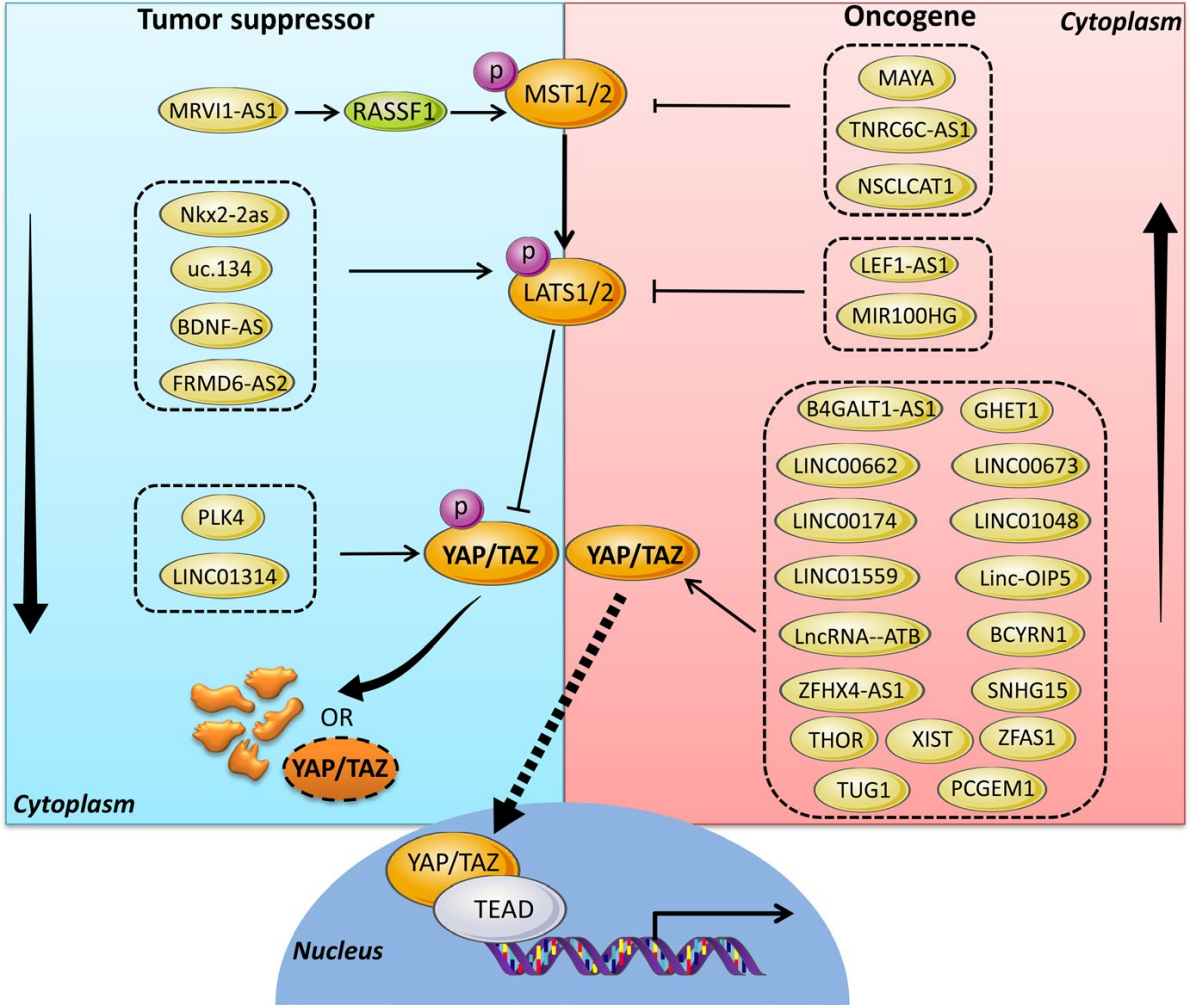
Links between lncRNAs and Hippo signalling cascade. Numerous lncRNAs have been demonstrated to be involved in cancer progression via regulating core components of the Hippo signalling pathway

Recently, lncRNAs are emerging as a critical mediator in a wealth of carcinogenic processes by targeting various downstream executors in Hippo signalling pathways (Figure 2). A number of lncRNAs, including B4GALT1 antisense RNA 1 (B4GALT1‐AS1),^35^ gastric cancer high expressed transcript 1 (GHET1)^36^ and X‐inactive specific transcript (XIST),^37^ were tightly associated with YAP to exert their functions in cancers. Zhang et al^35^ found that lncRNA B4GALT1‐AS1 was highly expressed in colon cancer cells by RNA‐seq. Depletion of B4GALT1‐AS1 repressed cancer cell colony formation and stemness. Further mechanism assay revealed that B4GalT1‐AS1 could directly bind to YAP. B4GALT1‐AS1 silencing could sequester YAP in cytoplasm and decrease YAP transcriptional activity, while overexpression of YAP attenuated the inhibition effect caused by B4GAlT1‐AS1 knockdown.^35^ In similar, another study showed that B4GALT1‐AS1 was expressed in osteosarcoma tissues as well as cell spheres at an enhanced level.^38^ Functionally, B4GALT1‐AS1 acted as an oncogene to enhance YAP mRNA stability and transcriptional activity by recruiting HuR, and in turn maintain osteosarcoma cells stemness, and promote migration and chemo‐resistance.^38^ Conclusively, these studies clarified an obvious association of B4GALT1‐AS1 and Hippo pathway, which may contribute to the malignant properties of tumour.^35^, ^38^ GHET1, located in chromosome 7q36.1, was firstly identified as an overexpressed lncRNA in gastric cancer.^39^ Guan ZB et al^36^ demonstrated an elevated expression of GHET1 in NSCLC and its knockdown could impede YAP expression, and thereby impair tumour cell proliferation, invasion ability and the epithelial‐to‐mesenchymal transition (EMT). XIST is a markedly elevated lncRNA in osteosarcoma tissues and cells.^37^ A panel of in vitro and in vivo studies confirmed that XIST knockdown restricted tumour cell growth, invasion and EMT. Interestingly, XIST acted as a decoy for miR‐195‐5p and thereby to alter YAP expression, implicating a regulatory role of XIST/miR‐195‐5p/YAP network in osteosarcoma progression.^37^

In addition to YAP, other components of Hippo cascade including TAZ, LATS1/2 and MST1/2 were also found involved in crosstalk with a variety of lncRNAs in carcinogenesis. For example, both LINC00174 and TAZ showed an upregulated expression pattern in human primary colorectal cancer (CRC) tissues as compared to corresponding normal tissues.^32^ Overexpression of LINC00174 or TAZ could enhance CRC cell proliferation motility. Bioinformatics and luciferase reporter assays revealed that LINC00174 may competitively bind to miR‐1910‐3p to increase TAZ expression in CRC carcinogenesis.^32^ MiR‐125a‐5p, an important endogenous tumour suppressor,^40^ was reported to target TAZ and inhibit EGFR pathway to repress retinoblastoma progression.^41^ A recent study performed by Yu et al^40^ suggested lncRNA BCYRN1 functioned as an oncogene by sponging miR‐125a‐5p to activate TAZ, and then results in cell proliferation, invasion and migration in glioma. In addition, Su et al^42^ demonstrated that mir‐100‐let‐7a‐2‐mir‐125b‐1 cluster host gene (MIR100HG), a well‐documented tumour facilitator in breast cancer^43^ and acute megakaryoblastic leukaemia,^44^ was also highly expressed in osteosarcoma. Functional assay and rescue experiments further confirmed that MIR100HG regulated cell proliferation, apoptosis and cell cycle mediated by epigenetically silencing LATS1/2 and inactivating Hippo pathway.^42^ Ras‐associated domain family member 1 (RASSF1) is a scaffold protein and functions as a tumour suppressor through regulation of cell cycle and apoptosis.^45^ LncRNA murine retrovirus integration site 1 homolog antisense RNA 1 (MRVI1‐AS1) was reported to be markedly downregulated in paclitaxel‐resistant cells and could promote RASSF1 expression to modulate MST1/2 and suppress downstream TAZ expression, and therefore increase nasopharyngeal cancer (NPC) chemo‐sensivitiy.^46^ In summary, these findings help to illuminate the role of lncRNA in the regulation of Hippo signalling to subsequently control cell proliferation and tumorigenesis.

### 3.2 LncRNAs induced by Hippo pathway

**Figure 3:**
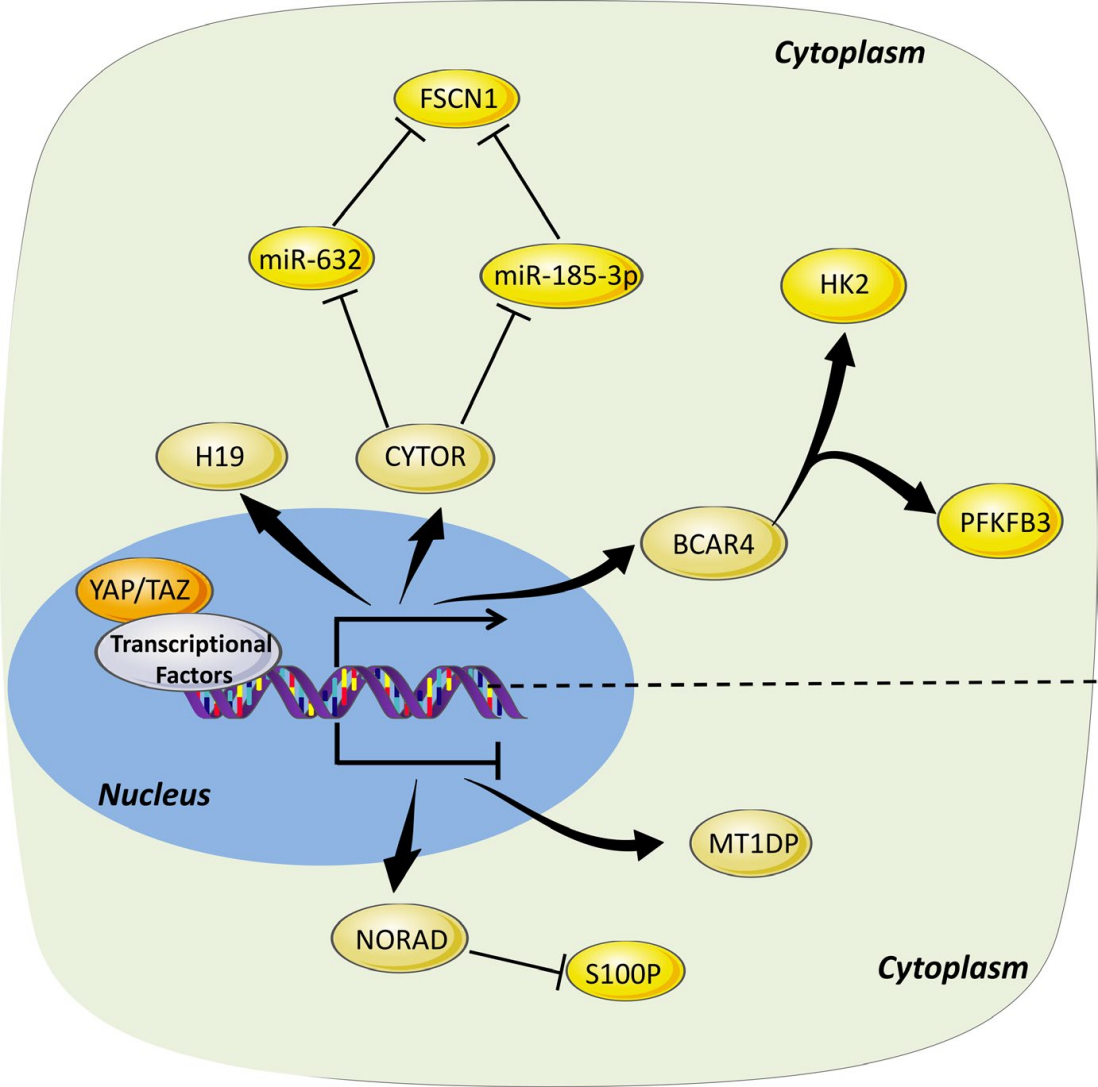
Links between lncRNAs and Hippo signalling cascade. Hippo signalling axis could modulate the transcriptional activity of certain lncRNAs and in turn play a critical role in cancers

Several studies demonstrated that the core components in Hippo pathways could also exert functions in the regulation of the expression as well as functions of lncRNAs, such as lncRNA breast cancer antiestrogen resistance 4 (BCAR4)^26^ and metallothionein 1D, pseudogene (MT1DP)^47^ (Figure 3). LncRNA BCAR4 is an upregulated lncRNA in multiple cancers with clinicopathological significance in prognosis.^4^ A study showed that BCAR4 and YAP expressions were positively correlated in breast cancer and closely associated with unfavourable recurrence‐free survival. Moreover, YAP could upregulate BCAR4 expression and coordinate the Hedgehog signalling pathway to promote the transcription of glycolysis activators HK2 and PFKFB3, and in turn to reprogramme glucose metabolism in breast cancer.^26^ LncRNA MT1DP, a tumour suppressor, could reduce cell proliferation and colony formation, while inducing the apoptosis in liver cancer.^47^ Alpha‐fetoprotein (AFP) is a well‐known biomarker in liver cancer progression and recurrence.^48^, ^49^ Functional assay suggested that MT1DP negatively regulated AFP by suppressing synthesis of Forkhead box A1 (FoxA1). Mechanistically, YAP and Runx2 together displayed an oncogenic activity by hindering lncRNA MT1DP in a FoxA1‐dependent manner in liver cancer.^47^ Other lncRNAs that are regulated by Hippo signalling pathway include cytoskeleton regulator RNA (CYTOR),^50^, ^51^ non‐coding RNA activated by DNA damage (NORAD)^52^ and H19.^53^ LncRNA CYTOR, also known as long intergenic ncRNA 00 152 (LINC00152), is located on chromosome 2p11.2 with a length of 828 nucleotides.^54^ CYTOR was found highly expressed in CRC compared with counterpart controls and proved to sustain proliferation and promote invasion and metastasis of cancer cells.^51^ CYTOR could be targeted and transcriptionally regulated by YAP and other Hippo pathway molecules in CRC cells, subsequently regulated fascin actin‐binding protein 1 (FSCN1) expression through sponging to miR‐632 and miR‐185‐3p, and thereby promoted the occurrence and metastasis of CRC.^51^ Besides, another study showed that NORAD, a unique kind of lncRNA that responds to DNA damage and maintains genome integrity and stability in cancers,^55^, ^56^, ^57^ was synergistically transcriptionally inhibited by the YAP/TAZ‐TEAD and the NuRD complex, which in turn affected the development and metastasis of lung and breast cancer via sequestration of S100P.^52^ Moreover, lncRNA H19, a well‐characterized oncogenic lncRNA in tumour progression, metastasis and chemo‐resistance,^58^, ^59^, ^60^ was also found abnormally expressed in osteosarcoma and could be upregulated by overexpression of YAP.^53^ To summarize, it is clear that Hippo pathway could intimately modulate certain lncRNA to engage in multiple processes of cancer development.

### 3.3 Reciprocal interaction between lncRNAs and Hippo pathway

**Figure 4:**
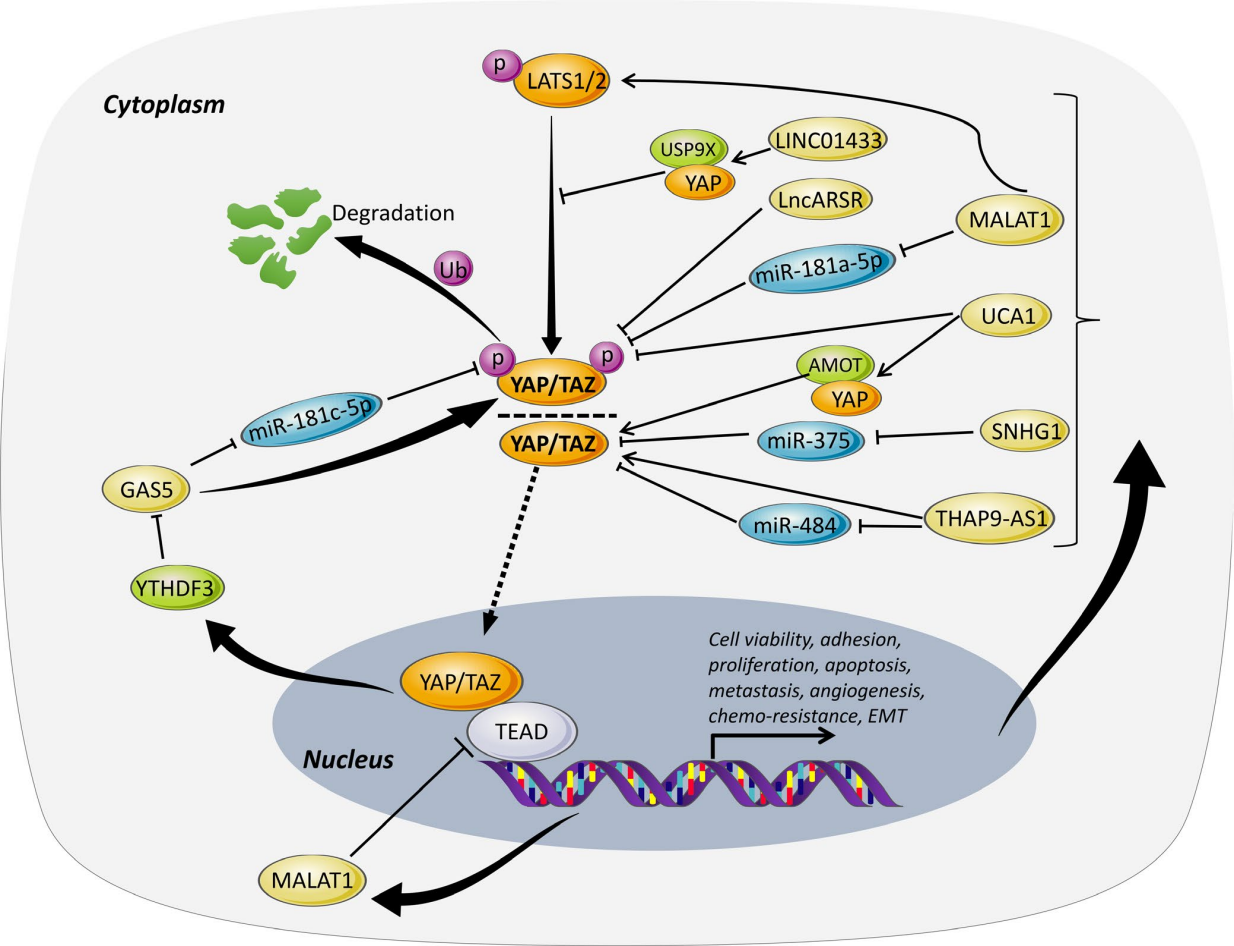
Reciprocal interaction between lncRNAs and Hippo cascade. A number of lncRNAs reciprocally interact with components of the Hippo signalling pathway to complete feedback loop in cancer progression

Of note, there are a number of lncRNAs show reciprocal feedback loop with Hippo signalling pathway, such as urothelial cancer‐associated 1 (UCA1),^61^, ^62^, ^63^, ^64^ growth arrest‐specific 5 (GAS5)^11^, ^65^ and metastasis‐associated lung adenocarcinoma transcript 1 (MALAT1)^66^, ^67^, ^68^ (Figure 4). UCA1 has displayed a trend of significantly increased expression in pancreatic cancer,^64^ thyroid cancer^61^ and ovarian cancer,^62^ when compared to adjacent normal tissue. Loss‐of‐function assay showed that UCA1 knockdown restrained cell proliferation and induced apoptosis, as evidenced by CCK‐8 and flow cytometry.^61^ Importantly, UCA1 could interplay with MOB1, LATS1 and YAP to form shielding composites, and thus suppress YAP phosphorylation to upregulate YAP expression. Moreover, UCA1 enhanced YAP nuclear localization and stabilization as well as increase TEAD luciferase activity. Besides, by using reverse‐phase protein array analysis and in vivo RNA antisense purification, Lin X and colleagues^62^ further identified that UCA1 could bind to a well‐known YAP regulator, angiomotin (AMOT) in ovarian cancer. Specifically, UCA1 enhanced AMOT‐YAP interaction to enhance YAP dephosphorylation and nuclear translocation.^62^ Interestingly, YAP could also promote expression of UCA1,^64^ indicating a reciprocal interaction between UCA1 and YAP that maintain the cancerous phenotype.

In addition, GAS5, a well‐acknowledged tumour suppressor, has been shown to exert essential inhibitory roles in cancer development and progression.^69^, ^70^ Gao et al^65^ reported that GAS5 was conspicuously downregulated and inversely correlated with miR‐181c‐5p expression in pancreatic cancer cells. Gain‐of‐function analysis showed that GAS5 dramatically dampened cell viability and antagonized the chemo‐resistance through regulation of miR‐181c‐5p to indirectly activate Hippo signalling.^65^ In addition, GAS5 was found to directly interplay with WW domain of YAP to facilitate YAP cytoplasmic localization in CRC.^11^ Moreover, GAS5 could trigger YAP^Ser127^ phosphorylation and promote ubiquitin‐mediated YAP degradation as an RNA scaffold.^11^ N6‐Methyladenosine (m^6^A) is the most abundant mRNA modification and plays a critical role in cancer progression.^71^ Currently, m^6^A‐modified lncRNAs in the regulation of YAP activation remain poorly defined.^11^, ^72^, ^73^ By using MeRIP‐seq and lncRNA‐seq, Ni et al^11^ further identified YAP could also target m^6^A reader YTHDF3, which reversibly bound m^6^A‐methylated GAS5 to facilitate its decay, suggesting a negative functional loop of GAS5‐YAP‐YTHDF3 axis in CRC progression.

Interestingly, lncRNA MALAT1, locating on human chromosome 11q13.1 with a transcript sequence of approximately 8 kb, is a context‐specific lncRNA among mammals that involved in the development of diverse malignancies by crosstalk with Hippo pathway.^74^, ^75^, ^76^, ^77^, ^78^, ^79^, ^80^ Early studies consistently showed that MALAT1 is highly expressed in cancerous tissue and facilitates tumour progression and metastasis in various cancers. For instance, a series of in vivo and in vitro experiments showed that MALAT1 knockdown can activate the Hippo cascade by upregulating miR‐181a‐5p, thereby hamper the proliferation and adhesion capacity of tumour cells in myeloma.^66^ In pancreatic cancer, MALAT1 showed extremely high expression pattern, leading to increased expression of YAP and decreased LATS1 expression, thus accelerating the tumour growth both in vitro and in vivo.^68^ In liver cancer, both YAP and MALAT1 were highly expressed, and YAP could increase MALAT1 expression at both transcriptional and post‐transcriptional levels.^67^ Serine‐/arginine‐rich splicing factor 1 (SRSF1) is a negative regulator of MALAT1. Importantly, YAP was reported to attenuate the nuclear retention of SRSF1 via interacting with AMOT and thereby abrogate the inhibitory effect of SRSF1 on MALAT1.^67^ Moreover, the combination of YAP overexpression and SRSF1 knockdown led to significantly enhanced tumour growth and migration.^67^ In contrast, a recent study by Kim *et al* demonstrated an opposite phenotype of MALAT1 in breast cancer.^81^ MALAT1 was obviously downregulated in breast cancer than parental tissue, and its level was negatively correlated with cancer progression and metastasis potential. MALAT1 acted as a tumour suppressor to impair cancer cell migration, invasion and metastasis by binding to and sequestering TEAD, and thereby blocking its association with co‐activator YAP.^81^, ^82^ In vivo assay using transgenic, xenograft and syngeneic models consistently showed a metastasis‐inhibitory role of MALAT1 in breast cancer.^81^ Hence, collectively, MALAT1 may form a positive bidirectional circuit with oncoprotein YAP in the regulation of cancer development and tumorigenesis in a cancer tissue‐specific manner. More comprehensive studies are therefore required to verify the oncogenic or tumour‐suppressive role in MALAT1 in cancers.^83^

Besides, other lncRNAs are also capable of forming feedback loops with Hippo pathway, such as LINC01433,^12^ small nucleolar RNA host gene 1 (SNHG1)^84^ and THAP9 antisense RNA 1 (THAP9‐AS1).^85^ As an oncogenic lncRNA, LINC01433 has been demonstrated to enhance tumour cell aggressiveness, including proliferation, migration, invasion and chemo‐resistance.^12^ Intriguingly, Zhang et al^12^ reported that LINC01433 stabilized YAP by upregulating the interaction between deubiquitinase USP9X and YAP and reduced YAP phosphorylation through inhibition of YAP‐LATS1 binding. Conversely, YAP could directly bind to LINC01433 promoter region to further activate its transcription.^12^ SNHG1 was reported to be remarkably upregulated in several types of human malignancies such as osteosarcoma and laryngeal squamous cell carcinoma (LSCC).^84^, ^86^, ^87^ SNHG1 knockdown obviously impeded tumour cell proliferation, migration and invasion, while induced apoptosis via participating in pleiotropic cancer‐related signalling pathways, such as Notch,^88^ Wnt/β‐catenin^87^ and Hippo pathway.^84^ Specifically, SNHG1 could serve as ceRNA to sponge to miR‐375 and thus promote YAP expression to regulate Hippo pathway in LSCC. Meanwhile, YAP could reversibly occupy promoter of SNHG1 to enhance its transcription, indicating a positive feedback regulation between SNHG1 and YAP.^84^ THAP9‐AS1 was found upregulated in pancreatic ductal adenocarcinoma (PDAC) tissues, and its expression was positively associated with YAP levels and remarkably correlated with worse clinical outcomes.^85^ THAP9‐AS1 exerted its pro‐carcinogenic role in PDAC both in vitro and in vivo by activating YAP. Notably, ectopic YAP expression could abolish the effects of THAP9‐AS1 knockdown, and vice versa.^85^ Mechanistically, THAP9‐AS1 could sponge miR‐484 to indirectly target YAP, or directly bind to YAP to result in upregulation of the expression and activity of YAP. Reciprocally, YAP/TEAD1 complex could enhance THAP9‐AS1 transcription to complete a feed‐forward loop.^85^

## 4 THE CLINICAL SIGNIFICANCE OF LNCRNAS INVOLVED IN HIPPO PATHWAY IN CANCERS

Detection of clinical biomarkers could enable early diagnosis of tumour, which is critical in clinical practice. Several core components of Hippo pathway have been implicated as potential biomarkers for prognosis and chemo‐resistance. For instance, YAP is found consistently elevated expressed in multiple cancers, such as osteosarcoma,^53^ breast cancer,^26^ liver cancer,^89^ bladder cancer,^90^ prostate cancer,^91^ pancreatic cancer^68^ and CRC.^51^, ^92^ YAP overexpression or increased activity may predict advanced tumour stages and poor clinical outcome in cancer patients.^25^, ^85^, ^92^ A more recent discovery indicated that expression of nuclear YAP (nYAP) was noticeably upregulated in docetaxel‐resistant prostate cancer cell lines than parental cells.^93^ Consistently, clinical data also revealed a higher nYAP level in the chemohormonal therapy (CHT) group than other groups, and patients with overexpressed nYAP in residual cancer after CHT predicted higher biochemical recurrence, indicating that nYAP level may be a promising prognostic factor in castration‐resistant prostate cancer patient treated with CHT.^93^ Furthermore, in conventional osteosarcoma, YAP/TAZ immune‐reactive score was significantly correlated with the overall survival (OS), and its nuclear expression was associated with progression‐free survival,^94^ suggesting a prominent link between YAP/TAZ expression and osteosarcoma prognosis.

Since the lncRNAs interacted with Hippo signalling pathway have a considerable impact on regulation of tumour cell functions, their clinical diagnostic and prognostic significances were also extensively delineated in studies. Some aberrantly expressed lncRNAs involved in Hippo pathway were found overtly correlated with prognosis outcomes and clinicopathological characteristics in cancers. For example, lncRNA‐ATB, a lncRNA activated by TFG‐β, was highly expressed in hepatocellular carcinoma (HCC) tissues compared to corresponding healthy liver samples.^95^ In HCC patients, expression of lncRNA‐ATB was positively associated with tumour size, TNM stage and unfavourable survival.^95^ A similar conclusion was drawn by Li et al that elevated H19 was associated with poor clinicopathological parameters.^90^ Inversely, lncRNA uc.134 was strikingly downregulated in HCC samples than adjacent tissues^96^ and its expression was positively associated with LATS1 and pYAP^S127^ levels in HCC and related to lymphatic metastasis and higher TNM stage. Moreover, HCC patients with lower expression level of uc.134 were apt to worsen OS.^96^ Similarly, downregulated expression of NORAD was also associated with lymph node metastasis (LNM) and poor prognosis.^52^ By contrast, lncRNA XIST was found markedly increased in osteosarcoma tissues and cell lines as demonstrated by qRT‐PCR,^97^, ^98^ and its expression was negatively associated with OS, and positively correlated with clinicopathological features, including larger tumour size, advanced Enneking stage, LNM and distant metastasis in osteosarcoma,^99^ suggesting XIST may be used as an independent clinical biomarker in osteosarcoma.^100^, ^101^, ^102^ Taken together, Hippo‐related lncRNAs appear to be innovative diagnostic and prognostic biomarkers for multiple cancers. However, there are still numerous challenges for their validation in clinical settings.^80^

## 5 THE THERAPEUTIC POTENTIAL OF LNCRNAS INVOLVED IN HIPPO PATHWAY

As mentioned above, the Hippo pathway comprises multiple downstream signalling proteins, such as YAP/TAZ, whose activation can endow cells with several hallmarks of cancer,^103^, ^104^ leading to uncontrolled cell growth, malignant transformation, acquisition of EMT and confer tumour cell resistance to chemo‐, radio‐ and even immunotherapy.^19^, ^30^, ^50^ Among them, chemo‐resistance remains a major obstacle to effective cancer treatment, contributing to metastatic progression and tumour relapse.^105^ As is shown, Mao et al^106^ demonstrated that SIRT1 enhances the interaction between YAP and TEAD4 to maintain cisplatin resistance In HCC. Another recent study confirmed that Hippo cascade also participated in osteosarcoma chemo‐resistance.^107^ Upon methotrexate/doxorubicin treatment, MST1 degradation increased, while LATS1/2 expression and YAP phosphorylation decreased in osteosarcoma cells. Further study revealed that activated nYAP subsequently resulted in transcription of downstream target genes, leading to cell proliferation and chemo‐resistance.^107^ Autophagy is an essential process implicated in tumour survival and chemo‐resistance.^95^, ^108^, ^109^ Wilkinson et al^110^ found that MST1/2 can phosphorylate LC3 and promoted cell autophagy, while decreased MST1 could constrain autophagy and thereby enhance cancer cell chemo‐sensitivity. Besides, EMT is a complicated process which may contribute to cytoskeletal remodelling and tumour cell migration and metastasis.^103^, ^111^ Shen et al conducted a study to show that TAZ and miR‐135b could form a positive feedback loop to modulate EMT process and metastasis in osteosarcoma.^112^ Hereto, researches on Hippo signalling cascade may improve our understanding with regard to a variety of tumour properties including, but not limited to, metastasis, chemo‐resistance and EMT. Therefore, targeting Hippo may be an attractive option for cancer therapy.^30^

Given the fact that lncRNAs are involved in cancer‐related signalling pathway to mediate tumorigenic process, it is therefore not surprising that these deregulated lncRNAs in Hippo cascade can also offer with the possibility as the attractive therapeutic candidates.^113^ Meanwhile, recent advances in biological drugs, such as antisense oligonucleotides (ASOs),^114^, ^115^ CRISPR/Cas9 to target lncRNAs, small interfering RNAs (siRNAs)^116^ and exosomal vectors, also implicate that lncRNAs could be used as prospective targets in cancer treatments.^111^ For instance, Liu et al^50^ found that CYTOR was among the most dramatically upregulated lncRNA in tamoxifen‐resistant breast cancer cells and in patient tissues with no response to tamoxifen treatment. CYTOR could activate Hippo and MAPK pathways via regulation of miR‐125a‐5p to enhance breast cancer cell survival upon tamoxifen treatment, indicating that targeting CYTOR may be a possible approach in reversing tamoxifen resistance in breast cancer.^50^

Furthermore, glucose metabolism plays a crucial role in promoting and maintaining tumour cell characteristics.^26^, ^117^ During glucose deprivation, AMPK could phosphorylate and inhibit YAP, and then the activated YAP enhances glucose consumption and lactate production to generate energy to support the tumour cellular activity,^26^ suggesting a role of Hippo pathway in promoting Warburg effect during carcinogenesis. A study by Zheng et al^26^ showed that BCAR4 acted as a downstream target of YAP‐dependent glycolysis. Of note, BCAR4 antisense‐locked nucleic acid could significantly abolish the YAP‐dependent glycolysis and tumorigenesis. Taken together, targeting YAP‐BCAR4‐glycolysis network may be a putative strategy for breast cancer treatment by reprogramming glucose metabolism.^26^ In addition, polo‐like kinase 4‐associated lncRNA (PLK4) is a downregulated lncRNA in HCC tissues and cell lines, and may serve as a tumour suppressor featured with YAP inactivation and subsequent cellular senescence induction.^118^ Talazoparib is a potent poly‐ADP‐ribosyl polymerase (PARP) inhibitor that can induce synthetic lethality in cancers with deleterious germline mutations in *BRCA*.^119^, ^120^ A very recent study reported that talazoparib could dramatically upregulate expression of PLK4 to show the tumour inhibitory effect in HepG2 tumour cells, which provides us with a novel pathway to target PLK4/YAP axis for the treatment of HCC.^118^ Certainly, the modulation of lncRNA/Hippo network may be an interesting and promising avenue for improvement of cancer treatment. However, lncRNA/Hippo‐based targeted therapy is still in its infancy and more experimental strategies as well as clinical trials are required in the near future.^80^

## 6 CONCLUSIONS AND PERSPECTIVES

Hippo pathway is one of the most complicated signalling pathways with multiple downstream effectors that respond to extracellular and intracellular stimuli to coordinately govern cell differentiation, migration and proliferation.^25^ Genetic or epigenetically provoked disruption of Hippo pathway leads to imbalanced regulation of these mechanisms, resulting in tumorigenesis.^25^, ^121^ Targeting Hippo signalling may provide novel approaches in treatment of cancer. However, given the fact that Hippo pathway has striking tumour regulatory activity in various contexts, the factors and concise regulation mechanisms for activation or inactivation of Hippo signalling are still poorly understood.^11^

LncRNAs are a subclass of ncRNAs with growing recognition for their role in diverse cellular activities. Altered expression and mutation of lncRNAs are reported to drive multifaceted cancer phenotypes by regulating gene expression and signalling pathways at various levels.^96^ Nowadays, a group of lncRNAs have been delineated to directly or indirectly target the core components of Hippo cascade, such as YAP, TAZ, LATS1/2 and MST1.^36^, ^58^, ^84^, ^122^ By contrast, Hippo can also modulate certain lncRNAs by affecting their transcriptional activity.^31^ The expression of lncRNAs is closely correlated with tumorigenesis and tumour aggressiveness. Importantly, lncRNAs related to Hippo signalling may be useful as predictive indicators for diagnosis and prognosis in cancers. Researches on the interaction between lncRNAs and Hippo signalling pathway may potentially offer us a more comprehensive understanding in cancer occurrence and progression.

However, it should be noticed that the link between lncRNAs and Hippo pathways may be cell type‐, context‐ and even tumour stage‐specific.^31^, ^52^ Thus, more studies are still warranted to further elucidate their detailed structures and functions for developing biomarker and individualized therapy.^80^ Besides the canonical Hippo pathway, there are studies reporting the non‐canonical Hippo signalling axis in the regulation of tumorigenesis.^123^, ^124^ Currently, the crosstalk between lncRNAs and non‐canonical Hippo pathway has not been elucidated yet, which may merit further exploration. Moreover, despite our understanding of lncRNA has been expanding in past decades, the discovery and functional annotation of lncRNAs still remain just the tip of an iceberg.^125^ Furthermore, in order to promote efficient therapeutic interventions in cancers by targeting lncRNAs and Hippo pathway, further in‐depth pre‐clinical and clinical studies are urgently needed.

## CONFLICTS OF INTERESTS

The authors have no conflicts of interest to declare.

## AUTHOR CONTRIBUTIONS

CT, KXY and JYH wrote the manuscript; LW, LQ, WCW, QL and ZHL reviewed and edited the manuscript before submission; CT and ZHL prepared the figures; and all authors read and approved the final version of the manuscript as submitted.

## Data Availability

The data that support the findings of this study are available from the corresponding author upon reasonable request.
